# Accelerated Aging in Cancer and Cancer Treatment: Current Status of Biomarkers

**DOI:** 10.1002/cam4.70929

**Published:** 2025-05-05

**Authors:** Soniya Abraham, Jay Parekh, Seohyuk Lee, Humayra Afrin, Mariya Rozenblit, Kim R. M. Blenman, Rachel J. Perry, Leah M. Ferrucci, Jessica Liu, Melinda L. Irwin, Maryam Lustberg

**Affiliations:** ^1^ Department of Internal Medicine Yale‐New Haven Health Bridgeport Hospital Bridgeport Connecticut USA; ^2^ Yale School of Medicine Yale University New Haven Connecticut USA; ^3^ Division of Medical Oncology, Yale School of Medicine Yale University New Haven Connecticut USA; ^4^ Department of Cellular & Molecular Physiology Yale School of Medicine New Haven Connecticut USA; ^5^ Yale School of Public Health Yale Cancer Center New Haven Connecticut USA

**Keywords:** accelerated aging, biomarkers of aging, cancer therapy toxicity, cellular senescence

## Abstract

**Background:**

Aging in humans is a heterogeneous process influenced by both biological and chronological factors. Biological age reflects an individual's physiological reserve and functional status. Increasing evidence suggests that cancer and its therapies accelerate biological aging. Many biomarkers have been evaluated to assess the biological age of patients with cancer. These biomarkers are emerging as potential tools to predict cancer‐related toxicity and an individual's functional capacity as well as to individualize treatment.

**Methods:**

This review summarizes the current literature on aging biomarkers in cancer patients, with a focus on markers of cellular senescence and epigenetic modification. We evaluate the existing evidence supporting their use as predictors of toxicity in patients undergoing chemotherapy and radiation therapy.

**Results:**

Biomarkers such as interleukin‐6 (IL‐6), leukocyte telomere length (LTL), and DNA methylation age show potential for assessing biological age, frailty, and functional reserve. The expression of p16INK4A has demonstrated promise in predicting therapy‐induced toxicity and making treating decisions. However, additional confirmatory studies are necessary to further validate these biomarkers before they can be utilized as decision aids.

**Conclusion:**

Aging biomarkers hold promise for individualizing cancer therapy and predicting treatment‐related toxicity. However, further studies are essential to validate their reliability and support their integration into clinical practice.

## 1 Introduction

Aging is a complex process and occurs at varying rates in individuals. Chronological age is the measure of time elapsed from an individual's birth to a given date, whereas biological age depends upon various biological and physiological factors such as genetics and comorbidities. Biomarkers of aging should differentiate between people of the same chronological age but different biological age [1, 2, 3]. According to the American Federation for Aging Research (AFAR) criteria, a true biomarker of aging must predict an individual's physiological, physical, and cognitive function, be feasible to test without causing harm, and be valid in both humans and laboratory animals [4].

Although aging is an established risk factor for the development of cancer, there is increasing evidence that cancer and cancer therapy itself can cause accelerated aging [5, 6, 7]. Studies have demonstrated increased morbidity and premature mortality in childhood cancer survivors [8, 9] and young adult cancer survivors [10]. Cancer survivors have a higher risk of developing secondary cancers and chronic health conditions compared to siblings of the same age [8, 9, 10, 11]. Various mechanisms have been proposed on how multimodal cancer treatments can cause accelerated biological aging. Chemotherapeutic agents and radiation can cause DNA damage and induce senescence in tumor cells as well as in nonmalignant cells [12]. In addition, these therapies can cause telomere shortening, epigenetic alterations, and exhaustion of stem cells. It is also shown that senescent cells secrete proinflammatory factors that may lead to the early onset of age‐related diseases and promote various aspects of tumorigenesis [12, 13, 14].

**FIGURE 1:**
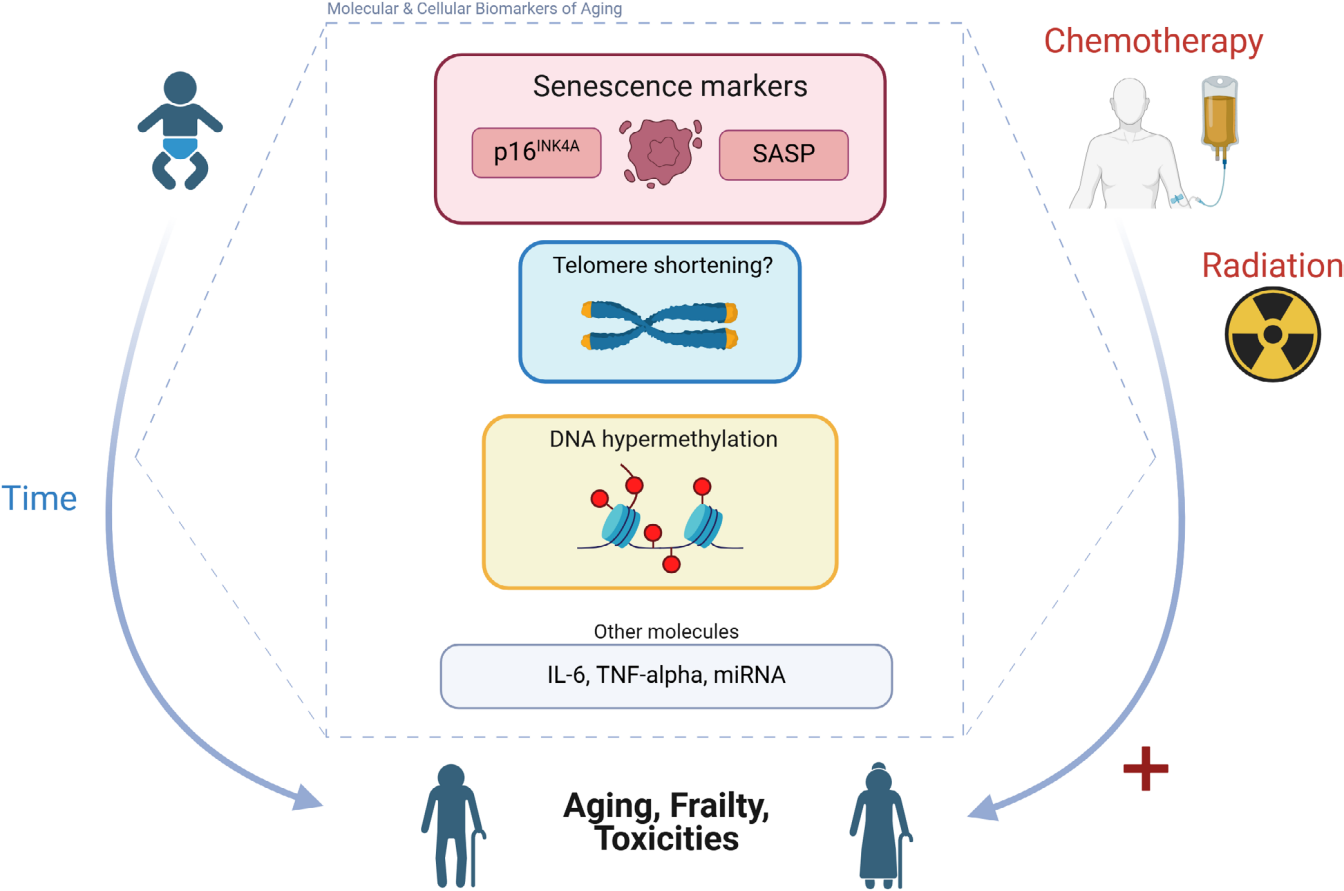
Intersection of phenotypical aging, carcinogenesis, cancer therapy, and toxicity. As phenotypic aging occurs at a macroscopic level, we have objective measurable biomarkers such as p16, DNA hypermethylation, and SASP. Evaluating these markers during cancer therapy helps identify the relationship between treatment and accelerated aging. Furthermore, assessing these markers can reveal associations between therapy‐induced aging and toxicity, enabling the downstream development of targeted strategies to mitigate treatment‐related adverse effects.

The number of cancer survivors has increased significantly due to advances in screening and better treatments [15, 16, 17]. Unfortunately, chemotherapy as well as other cancer treatments including radiotherapy and immunotherapy are associated with significant toxicities. These toxicities can impair quality of life, impact dose intensity, and even lead to early discontinuation of therapy. Attempts are ongoing to develop tools to predict the development of toxicity and permit earlier shared decision‐making to determine the tolerability of cancer therapy [18, 19, 20, 21, 22]. Oncologists widely use risk calculators to make treatment decisions; however, these are not precise tools to predict individual tolerance and toxicity development to various cancer therapies [23, 24]. Many biomarkers have been studied to assess the biological age and to predict the development of future toxicity among patients receiving therapy for cancer [25] (Figure 1).

In this review, we summarize the available evidence on molecular and cellular biomarkers of aging in patients with cancer and their associations with cancer treatment‐related toxicity.

## 2 Methodology

**FIGURE 2:**
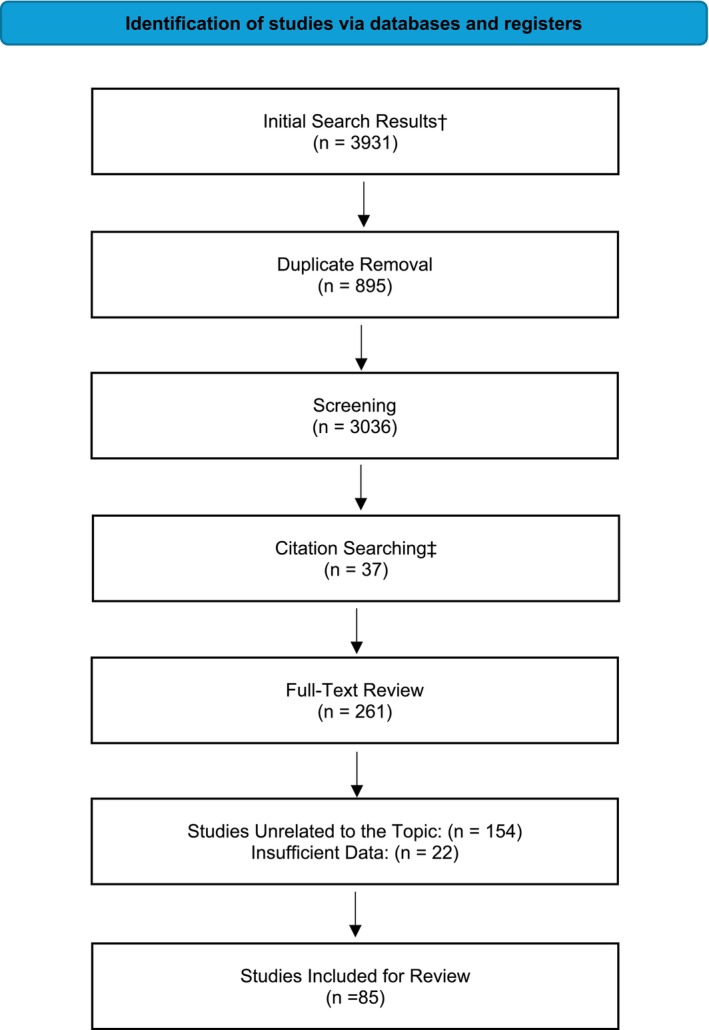
Flow diagram of study selection process. ^†^Records identified through database searching (MEDLINE, Scopus, Web of Science, Cochrane Library). ^‡^Records identified from citation searching from relevant reviews. Page MJ, McKenzie JE, Bossuyt PM, Boutron I, Hoffmann TC, Mulrow CD, et al. The PRISMA 2020 statement: An updated guideline for reporting systematic reviews. *BMJ* 2021;372:N71. doi: 10.1136/bmj.n71.

A comprehensive search was performed to select relevant studies available up to August 31st 2024. Databases queried included MEDLINE, Scopus, Web of Science, and Cochrane Library. Abstracts from several national meetings, including the American Society of Clinical Oncology, American Society of Hematology, American Association of Cancer Research, and European Society of Medical Oncology were also selected. The following Medical Subject Headings (MeSH) and keywords were used in the search: Biomarkers of aging, Cellular Senescence, Cancer therapy, Toxicity, Accelerated Aging, Epigenetic aging, Epigenetic clocks, DNA methylation, Telomere, Telomerase, Cancer survivorship, Senescence associated secretory phenotype, and p16^INK4A^. Preclinical and clinical trials, studies evaluating the association of aging, biomarkers related to aging, and cancer treatments were included. Initially, 3931 publications were retrieved. Duplicate studies, studies unrelated to the topic, publications without English language translations, and insufficient data were excluded from the review. Additional studies were independently selected from the reference lists of included papers. After identifying studies through manual and keyword searches, citation searches were conducted to evaluate studies citing them for inclusion. Ultimately, 85 publications were included in this review (Figure 2).

## 3 Biomarkers in Chemotherapy Exposure

### 3.1 P16^INK4A^

There is compelling evidence demonstrating an increase in p16^INK4A^ with chemotherapy. In a study of 63 patients, p16^INK4A^ expression was measured in T lymphocytes pre‐ and post‐stem cell transplantation. Higher p16^INK4A^ expression correlated with a history of exposure to chemotherapy and post‐transplant status [26]. In a prospective study involving 146 women with Stage I–III breast cancer, p16^INK4A^ expression was measured pre‐ and post‐chemotherapy. A significant increase in p16^INK4A^ expression was noted in patients who received chemotherapy. The increase was significantly greater in patients who had low baseline p16^INK4A^ expression. Anthracycline‐based chemotherapy regimens were found to be more pro‐aging compared to non‐anthracycline‐based regimens [27]. Another study of young adult cancer survivors found to have higher p16^INK4A^ expression in cancer patients compared to age‐matched controls, and this increase was equivalent to 25 years of chronological aging. This same study also demonstrated a dramatic increase in p16^INK4A^ levels pre‐ and post‐chemotherapy [28].

A prospective study involving 33 women with stage I–III breast cancer assessed the levels of senescence markers, senescence associated cytokines, and leucocyte telomere length (LTL), before initiation and prospectively till 12 months after completion of anthracycline‐based chemotherapy. An exponential increase in peripheral blood T lymphocytes p16^INK4A^ expression was noted after adjuvant chemotherapy, equivalent to 14 years of chronological aging. Additionally, there was an increase in the expression of Alternating Reading Frame (ARF), VEGFA, and Monocyte Chemotactic Protein 1 (MCP1). There was no significant increase in LTL, IL‐7, IL‐8, and IL‐6 levels. This study also assessed aging biomarkers in 176 long‐term breast cancer survivors, and p16^INK4A^ expression was higher in women treated with chemotherapy compared to women not treated with chemotherapy, suggesting that the increase in p16 expression can last for at least several years [29]. Another study demonstrated that doxorubicin induced a DNA damage response in human cardiac progenitor cells, resulting in telomere shortening and premature expression of senescence marker p16^INK4A^ [30].

Two studies involving multiple myeloma patients who underwent autologous hematopoietic stem cell transplant (AHSCT) revealed that prior to any treatment, the expression of p16 did not differ when compared to age‐matched controls. However, a significant increase in p16 expression was observed after therapy, including AHSCT, indicating the impact of cancer therapy on p16^INK4A^ [26, 31].

### 3.2 Inflammatory Biomarkers

A study discovered that breast cancer survivors who underwent surgery, chemotherapy, and radiation treatment have higher levels of tumor necrosis factor‐α and IL‐6, as well as increased pain and comorbidity burden compared to their peers of the same age who did not have cancer. These effects were not present before the cancer treatment but were observed 18 months after treatment [32]. Brouwers et al. studied the relationship of biomarkers of aging with chronologic age and clinical frailty in non‐metastatic breast cancer patients. They demonstrated IL‐6 levels correlated with age and frailty in elderly cancer patients [33]. Later, they conducted a prospective observational study involving 109 women over 70 years of age with early breast cancer to assess the impact of adjuvant chemotherapy on biological and clinical aging and frailty [34]. Subjects were enrolled after the surgery and were assigned to the adjuvant chemotherapy group or the control group. Geriatric assessment and quality of life (QoL) were evaluated and levels of aging biomarkers were measured at inclusion, after 3 months, and 1 year. There was no significant change in IL‐6 levels at the end of 1 year in the chemotherapy group compared to controls and LTL decreased comparably in both groups. Interestingly, there was a significant increase in TNF‐α and MCP‐1 and a decrease in IL‐10 and IGF‐1 from baseline in the chemotherapy cohort, suggesting age acceleration.

In a study involving 160 metastatic colon cancer patients receiving palliative chemotherapy, researchers found that elevated CRP and IL6 levels before starting chemotherapy were independently associated with shorter overall survival (OS) and progression‐free survival (PFS). This suggests that these markers could be helpful in daily clinical practice, providing valuable information that could facilitate discussions on prognosis and support decision‐making [35].

### 3.3 Telomere Length and Telomerase Activity

Telomere length and telomerase activity have been studied extensively in relation to aging and carcinogenesis. Telomerase, a ribonucleoprotein enzyme complex with core components including telomere reverse transcriptase (TERT), plays a vital role in the maintenance of telomere length. There is substantial evidence demonstrating that the lack of adequate telomerase activity leads to progressive telomere shortening and the expression of senescence associated secretory phenotype (SASP) in cells, making it an interesting area for evaluation as a potential biomarker of aging [36].

Lee et al. noted telomere shortening in Non Hodgkins Lyphoma patients after chemotherapy [37]. In a study conducted by Yoon et al., 32 patients with various solid tumors showed significantly shorter telomeres in their peripheral blood mononuclear cells (PBMCs) after undergoing multiple chemotherapy cycles [38]. Additionally, Unryn et al.'s study on 20 head and neck cancer patients reported a decrease in telomere length over time [39]. Another study demonstrated that patients who received chemotherapy and/or radiation after surgery had higher levels of DNA damage and lower telomerase activity in peripheral blood compared to patients who underwent surgery alone on long‐term follow‐up. However, there was no significant difference in telomere length following treatment [40]. A cross sectional study done in early stage breast cancer patients who were followed 3–6 after treatment. Leucocyte DNA damage, Telomerase activity, telomere length and inflammatory marker sTNF RII were measured. Higher DNA damage and lower telomerase activity were associated with lower executive functions. Lower telomerase activity was also associated with worse attention and motor speed [41].

A systematic review by Gallichio and colleagues identified 25 studies on the effect of cancer treatment on telomere length. They found inconsistent results comparing the association between change in telomere length and cancer treatment in solid and hematological malignancies [42]. Although several studies showed a significant association between cancer and a decrease in telomere length in PMBC [37, 38, 39], two studies on hematological cancer patients showed elongation of telomere length [43, 44]. There is limited data available to explain the disparities in the findings. These inconsistencies may be attributed to different types of treatments affecting telomere length in varying ways, or some treatments not affecting telomere length at all. Therefore, further studies are needed to investigate the reliability of telomere length changes [42].

Overall, chemotherapy has been associated with the upregulated expression of various cytokines such as p16^INK4A^, ARF, VEGF‐A, and MCP1, and potentially shortening telomere lengths. This evidence supports that cancer treatment may create a pro‐aging milieu that may lead to worse outcomes. Further research is needed to elucidate the impacts these changes may have.

## 4 Biomarkers in Radiation Exposure

There has been growing evidence that radiation exposure as a therapeutic intervention leads to several long‐term side effects. Wang et al. demonstrated the effect of radiation in murine models with respect to the induction of senescence [45]. In this study, the authors also noted that mice exposed to sub‐lethal doses of radiation also had increased expression of biomarkers of cellular senescence, which included p16^INK4A^. Subsequently, another study not only confirmed this but demonstrated that murine models exposed to ionizing radiation had long‐term expression of senescence markers in vivo [46]. Research in humans has found that childhood cancer survivors who underwent skin biopsies noted that childhood cancer survivors exposed to therapeutic interventions including radiation had higher p16^INK4A^ expression in skin biopsies of the scalp compared to skin biopsies of an area not exposed to radiation [47]. These data support the hypothesis that the effects of radiation therapy, unlike chemotherapy related to cellular senescence, may be limited to the exposed areas [12].

In human mammary fibroblasts in vitro models, exposure to radiation leads to dose‐dependent increased production of beta galactosidase and leads to G1 cell cycle arrest [48]. Meanwhile, radiation‐induced vascular injury has been established using several biomarkers, such as pro‐BNP, CRP, troponins, and fibrinogen, but they have not been established as markers of senescence in this population [49]. In murine models, exposure to ionizing radiation induced senescence in hematopoietic stem cells, which was demonstrated by increased expression of p16^INK4A^ and beta galactosidase [45]. Similar results were also found in mice [46]. Increased p16^INK4A^ expression was also demonstrated in lung tissue exposed to radiation in mice models [50]. In breast cancer patients exposed to radiation therapy, it was demonstrated that the low radiation‐induced lymphocyte apoptosis phenotype was associated with enriched cellular senescence pathways such as cell cycle/NF‐κB, G‐protein‐coupled receptors, and interferon signaling [51]. Telomere shortening in bone marrow mononuclear cells was noted after high‐dose radioimmunotherapy myeloablative conditioning with ^90^Y‐ibritumomab tiuxetan in Non Hodgkin's lymphoma patients [52].

## 5 Biomarkers With Epigenetic Modification in Cancer Therapy Exposure

Recent evidence suggests that epigenetic modifications, especially DNA methylation, are associated with age and age‐related disorders [53]. Epigenetic age is determined using DNA methylation and is strongly associated with chronological age in human studies. Epigenetic age acceleration (EAA) occurs when an individual's epigenetic age is greater than chronological age. Epigenetic clock refers to the hypothetical possibility of the existence of a biological clock that correlates based on changes that occur due to DNA methylation [54, 55].

DNA methylation age was assessed using the Levine epigenetic clock in 133 head and neck cancer patients without metastasis. EAA was noted immediately after, at 6 months, and at 1‐year postradiation therapy, with EAA being most pronounced immediately after radiation [56].

Sehl et al. studied the effect of radiation and chemotherapy on markers of EAA in breast cancer patients who had undergone surgery but had yet to start adjuvant therapy. Four epigenetic age measures—intrinsic, extrinsic, phenotypic, and Grim were—calculated using DNA methylation profiles. Significant EAA was seen in Grim, extrinsic, and phenotypic algorithms, with a more pronounced increase in patients undergoing radiation alone [57]. Similarly, Gilmore et al. compared peripheral blood epigenetic age before and within 1 month after completion of adjuvant/neoadjuvant chemotherapy in stage I–III breast cancer patients compared to matched noncancer controls. DNA methylation was measured and epigenetic age was calculated using seven epigenetic algorithms or clocks. Four epigenetic clocks (Hannum, Phenotypic, Grim, and Extrinsic) showed EAA after chemotherapy, whereas the Horvath and Skin blood algorithm showed epigenetic age deceleration. Except for the phenotypic algorithm, no significant difference was noted in epigenetic age prior to chemotherapy when compared to controls. The phenotypic age of the case cohort was 2 years older than the controls pre‐chemotherapy, and the biological or epigenetic age increased four times after chemotherapy [58].

A large multicenter study discovered that older breast cancer survivors, especially those who had chemotherapy, exhibited increased epigenetic aging compared to controls. The survivors were biologically 1.04–2.22 years older than controls approximately 24–36 months after systemic therapy on the basis of Horvath, EEA, GrimAge, and DunedinPACE measures, and the difference in biological age was even greater, ranging from 1.97 to 2.71 years in patients who received chemotherapy. In addition, older epigenetic age was associated with worse self‐reported cognition [59].

Higher EAA was also noted in testicular cancer survivors treated with cisplatin‐based regimes when compared to survivors treated with surgery alone [60]. In ovarian carcinoma, a study investigated the potential of interindividual methylation variation in WBC DNA for prognostic value and PFS in relation to paclitaxel treatment. High‐level methylation within the ESR1 gene was related to CA125 response and neuropathy in the paclitaxel group [61]. EAA was identified in pediatric medulloblastoma survivors and was associated with neurocognitive impairments [62]. EAA has also been noted in childhood cancer survivors and was significantly associated with various chronic health conditions [63]. Collectively, these studies demonstrate that significant epigenetic acceleration occurs following cancer treatment.

## 6 Biomarkers With Cancer Therapy‐Associated Toxicities

Numerous studies have largely consistently reported significant associations between biomarkers of aging and cancer therapy‐associated toxicities. A prospective multicentre study including 152 early breast cancer patients receiving taxane chemotherapy found that the development of chemotherapy‐induced peripheral neuropathy (CIPN) was associated with chemotherapy‐induced increases in p16 expression [64]. This study also identified that patients with elevated p16 expression prior to chemotherapy were at higher risk of developing CIPN. Intensive chemotherapy and the development of grade III–IV hematologic toxicities have been associated with increased levels of p16^INK4A^ and ARF [29]. Research has suggested that chemotherapy‐induced fatigue is more common in breast cancer patients with higher baseline p16^INK4A^ expression [65]. Additionally, higher p16^INK4A^ and high sensitivity CRP levels were associated with lower exercise capacity in childhood cancer survivors [66].

Paclitaxel is a commonly used chemotherapeutic agent in breast cancer [67]. However, it can cause cumulative toxicity, which can compromise long‐term administration. Preclinical evidence suggests that the burden or percentage of critically short telomeres (< 3 kilobases), but not the average length of telomeres, is associated with weekly paclitaxel toxicity [68]. Hodgkin's lymphoma patients who developed secondary cancers after chemotherapy were found to have shorter pre‐treatment telomere length and complex chromosomal rearrangements compared to the patients who did not develop secondary cancer [69]. Although several studies have demonstrated an association between baseline telomere length and chemotherapy‐related toxicities [68, 69, 70], others have reported no such relationship [71, 72].

IL6, IL8, TNF‐α, Monocyte Chemoattractant protein 1, and IGF binding proteins are some proteins associated with SASP, a complex occurrence of several events at the cellular level suggestive of cellular aging. Although SASP can contribute to tumor suppression, SASP has been frequently associated with chronic low‐grade inflammation playing the classic role of a dual‐edged sword with respect to carcinogenesis [73]. Xia et al. demonstrated that continuous doxorubicin therapy resulted in an increased amount of SASP in cardiac muscle cells, often leading to cardiac senescence and therefore, cardiotoxicity [74]. Increased EAA has also been associated with fatigue and elevated inflammatory markers (IL6, CRP) among head and neck cancer patients undergoing radiotherapy [56]. In contrast, Brouwers et al. found in their cohort of patients with early breast cancer randomized following surgery to either chemotherapy with docetaxel and cyclophosphamide or no chemotherapy, neither leukocyte telomere length nor levels of IL‐6, IL10, TNF‐α, IGF‐1, MCP‐1, and RANTES were associated with the development of Grade II or higher toxicity [34]. In a prospective study, 400 women aged 60 years or older with primary breast cancer (stage 0–III) were compared with 329 frequency‐matched controls. The participants were monitored annually for 60 months, and blood samples were collected during each assessment from 2016 to 2020. The study found that survivors had significantly higher levels of IL‐6 compared to controls before receiving systemic therapy and at 12, 24, and 60 months. Additionally, elevated levels of IL‐6, IL‐10, and TNF‐alpha were linked to lower scores on attention, processing speed, and executive function tests [75].

These developments have led to the exploration of potential therapeutics to address SASP in association with cancer therapy and its role in aging and toxicity mitigation. A novel class of drugs identified as senolytics is also under exploration with respect to the tumor microenvironment (TME) as senescent cells also play an essential role in modulating tumor response to anticancer therapy [76]. Drugs such as navitoclax and dasatinib have been evaluated to play a role in clearing senescent cells. Navitoclax, a regulator of the apoptosis pathway, has been found to eliminate senescent cells in the TME. Preclinical trials have identified a role for senolytics in the mitigation of carcinogenesis and other age‐related comorbidities besides playing a role in alleviating complications of radiation and cancer treatment. Clinical trials of dasatinib plus quercetin, a combination frequently evaluated for its role in senolysis to address frailty in post‐bone marrow transplant survivors and childhood cancer survivors, have been conceptualized to address this area further [77].

Evidence suggests that miRNA biomarkers of aging, including polymorphisms of miR‐605, miR‐5197, miR‐27a, and miR‐146a, can be associated with chemotherapy toxicity in patients with lung cancer. miR‐5197 rs2042253 was associated with overall severe toxicity and hematological toxicity in lung cancer patients treated with cisplatin‐based chemotherapy. Subgroup analysis demonstrated that polymorphism rs2910164 of miR‐146a was associated with severe hepatotoxicity in male smokers. In addition, polymorphism rs895819 of miR‐27a showed significant gastrointestinal toxicity in ages > 56 years both smokers and non‐smokers. This suggests that MiRNA polymorphisms may act as a predictive tool for the evaluation of toxicity in platinum‐based chemotherapy in lung cancer patients, if further validated in other studies [78]. Overexpression of miR‐27 is a significant predictive factor for fluoropyrimidine toxicity [79]. rs895819A>G in miR‐27a may be clinically important for risk stratification of fluoropyrimidine toxicity in DPYD risk variant carriers, also pending further validation.

A recent study found a relationship between p16 INK4A and the epigenetic clocks Hannum and PhenoAge, as well as multiple mRNA markers of T cell senescence. The study discovered that molecular markers of aging, especially PBTL p16 and PhenoAge, have the potential to predict frailty in older adults with hematological malignancies [80].

In summary, there is increasing evidence for biomarkers of aging as a promising screening tool to possibly predict post‐chemotherapy toxicities pending additional confirmation and validation studies. Biomarkers such as IL‐6, LTL, and DNA methylation age can be potentially utilized to evaluate a patient's biological age, frailty, or functional reserve in future studies. The expression of p16INK4A shows promise as a biomarker for making treatment decisions and predicting cancer therapy‐induced toxicity. However, additional confirmatory studies are necessary to further validate these biomarkers before they can be utilized as decision aids.

## 7 Future Directions

In the recent decades, there has been an effort to identify numerous biomarkers of aging, as evidenced above. However, the search for an ideal biomarker, especially for use in cancer patients, is an ongoing area of investigation. Although p16^INK4A^ has demonstrated robustness in terms of evidence of cellular aging, there is not enough evidence to quantify its relationship with intensity and severity of aging in association with exposures including anticarcinogenic therapeutics. P16^INK4A^ expression and its association with aging vary between tissue types, making this an important area of further investigation to determine appropriateness as a biomarker [81, 82]. Similarly, telomere length and telomerase activity have demonstrated variability as well. A study found a decline in telomerase activity in PBMCs with age and negligible activity in some older adults, raising the potential question of reliability warranting further investigation [83]. Epigenetic markers share several such concerns. A study conducted multiplex mass cytometry analysis and profiled chromatin modifications at the single‐cell level and found aging was associated with increased heterogeneity between individuals and increased cell‐to‐cell variability in chromatin modifications [84]. Similarly, a study raising concerns regarding DNA methylation clocks as a marker has been conducted with potential for low accuracy [85].

An ideal biomarker would be cheap, easily detectable, and would not only have predictive information but would also provide information regarding prognostication with clinical applications. In the years to come, consistent effort should be made to identify and validate the ideal biomarker, as well as subsequently design intervention studies that evaluate whether accelerated aging due to cancer therapies can be mitigated. The scope for clinical applications of aging biomarkers is vast. A growing body of data are accumulating regarding aging biomarkers and their association with chemotherapy and radiation‐related toxicity. Although the ability to identify and predict toxicity to improve outcomes is promising, there is a need for further validation of these data. Additional research is critical to solidify the current evidence such that it can be applied clinically and to personalize care.

## Author Contributions

**Soniya Abraham:** conceptualization (supporting), writing – original draft (lead), writing – review and editing (equal), methodology (equal), project administration (lead). **Jay Parekh:** writing – original draft (supporting), methodology (equal), writing – review and editing (equal), visualization (equal). **Seohyuk Lee:** methodology (equal), writing – review and editing (equal). **Humayra Afrin:** methodology (equal), writing – review and editing (equal). **Mariya Rozenblit:** writing – review and editing (equal). **Kim R. M. Blenman:** writing – review and editing (equal). **Rachel J. Perry:** writing – review and editing (equal). **Leah M. Ferrucci:** writing – review and editing (equal). **Jessica Liu:** visualization (equal), writing – review and editing (equal). **Melinda L. Irwin:** writing – review and editing (equal). **Maryam Lustberg:** conceptualization (lead), writing – review and editing (equal), supervision (lead), resources.

## Ethics Statement

The authors have nothing to report.

## Conflicts of Interest

The authors declare no conflicts of interest.

## Data Availability

The authors have nothing to report.
